# High-dose dobutamine stress SSFP cine MRI at 3 Tesla with patient adaptive local RF shimming using dual-source RF transmission

**DOI:** 10.1186/1532-429X-14-S1-P252

**Published:** 2012-02-01

**Authors:** Alexander Berger, Bernhard Schnackenburg, Christopher Schneeweis, Sebastian Kelle, Christoph Klein, Marc Kouwenhoven, Eckart Fleck, Rolf Gebker

**Affiliations:** 1 Department of Internal Medicine/Cardiology, German Heart Institute Berlin, Berlin, Germany; 2 Philips Healthcare, Hamburg, Germany; 3 Philips Healthcare, Best, Netherlands

## Background

Image quality of cine imaging using steady state free precession (SSFP) sequences at 3T is insufficient due to increased RF-inhomogeneity (B1 field) and the high sensitivity of SSFP sequences to off-resonance artefacts. Recently, the introduction of a dual source RF transmission system with patient-adaptive local RF-shimming has led to a significant improvement of image quality of SSFP imaging at 3T.

The objective of this study was to prospectively evaluate the feasibility, image quality and diagnostic accuracy of high-dose dobutamine stress magnetic resonance imaging (DSMR) at 3T comparing dual-source versus single-source transmit technology.

## Methods

DSMR was performed in 44 patients with each participant undergoing cine imaging at rest and during dobutamine infusion using both dual- and single-source transmit technology.

B1-maps and measurements of contrast to noise ratio (CNR) were evaluated to quantify the effect of RF calibration in both transmission modes.

Analysis of image quality (0=non diagnostic, 1=severe artifact, 2=slight artifact, 3=no artifact) and wall motion was performed at rest and at maximum stress comparing single- and dual-source technology.

CAD was defined on invasive coronary angiography as the presence of ≥70% stenosis.

## Results

The mean percentage of the intended flip angle within the heart increased from 88% ± 9.1 with single-source to 103% ± 5.6 with dual-source (p<0.001). Deviation of the flip angle from the base to the apex along the pseudo-long axis decreased from 29.8% ± 12.9% with single-source to 12.8% ± 7.2% with dual-source.

CNR increased for dual-source vs. single-source especially pronounced at the apex (63.4 ± 24.2 vs. 36.5 ± 16.5, p<0.001) but also at the base (50.1 ± 14.8 vs. 39.3 ± 15.8, p<0.001).

**Figure 1:**
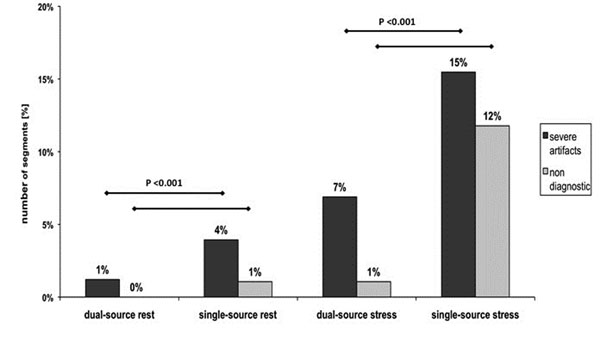
Analysis of image quality of DSMR SSFP cine imaging at 3 Tesla. A significant difference existed between the number of segments with severe artifacts and no diagnostic segments comparing single-source and dual-source transmit technology both at rest and even more pronounced at maximum stress.

Image quality of dual-source was higher than single-source both at rest (2.8 ± 0.5 vs. 2.6 ± 0.7, p<0.001) and stress (2.5 ± 0.7 vs. 2.0 ± 1.0, p<0.001). The number of segments with either severe artifacts or non-diagnostic image quality at stress was 27% using single-source compared to only 8% using dual-source (figure 1).

**Figure 2:**
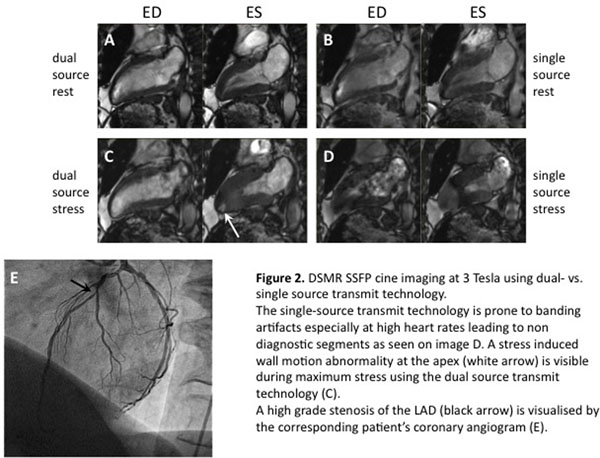


No significant differences between dual-source DSMR and single-source DSMR were seen regarding sensitivity (92% vs. 83%, p=0.38) and specificity (88% vs. 50%, p=0.25) due to the relatively small patient cohort. Diagnostic accuracy of dual-source DSMR (90%) was significantly higher than single-source DSMR (77%) (p=0.006) (figure 2).

## Conclusions

We demonstrated that using a dual-source transmit technology in a standard DSMR protocol is feasible in a 3T environment. Furthermore, the dual-source transmit technology provides better image quality and higher diagnostic accuracy compared to single-source transmit technology.

## Funding

None.

